# Ocean acidification drives community shifts towards simplified non-calcified habitats in a subtropical−temperate transition zone

**DOI:** 10.1038/s41598-018-29251-7

**Published:** 2018-07-27

**Authors:** Sylvain Agostini, Ben P. Harvey, Shigeki Wada, Koetsu Kon, Marco Milazzo, Kazuo Inaba, Jason M. Hall-Spencer

**Affiliations:** 1 Shimoda Marine Research Center, University of Tsukuba, 5-10-1 Shimoda, Shizuoka, 415-0025 Japan; 2 Dipartimento di Scienze della Terra e del Mare, University of Palermo, CoNISMa, via Archirafi 28, 90123 Palermo, Italy; 3 Marine Biology and Ecology Research Centre, University of Plymouth, Plymouth, PL4 8AA UK

## Abstract

Rising atmospheric concentrations of carbon dioxide are causing surface seawater pH and carbonate ion concentrations to fall in a process known as ocean acidification. To assess the likely ecological effects of ocean acidification we compared intertidal and subtidal marine communities at increasing levels of *p*CO_2_ at recently discovered volcanic seeps off the Pacific coast of Japan (34° N). This study region is of particular interest for ocean acidification research as it has naturally low levels of surface seawater *p*CO_2_ (280–320 µatm) and is located at a transition zone between temperate and sub-tropical communities. We provide the first assessment of ocean acidification effects at a biogeographic boundary. Marine communities exposed to mean levels of *p*CO_2_ predicted by 2050 experienced periods of low aragonite saturation and high dissolved inorganic carbon. These two factors combined to cause marked community shifts and a major decline in biodiversity, including the loss of key habitat-forming species, with even more extreme community changes expected by 2100. Our results provide empirical evidence that near-future levels of *p*CO_2_ shift sub-tropical ecosystems from carbonate to fleshy algal dominated systems, accompanied by biodiversity loss and major simplification of the ecosystem.

## Introduction

Rising atmospheric concentrations of carbon dioxide are causing surface seawater pH and carbonate ion concentrations to fall in a process known as ocean acidification^1^. Marine organisms are expected to differ widely in their responses to this increase in CO_2_ levels; partially due to the fact that altered carbonate chemistry stresses many organisms (e.g. negative effects on calcification, reproduction, feeding rate and early life-stage survival^2^), but is a resource for others (e.g. enhanced primary production and carbon fixation rates^3^). Most studies have focussed on the effects of ocean acidification on isolated organisms, yet these experiments seldom include community and ecosystem-level interactions and so it is difficult to assess how the results apply to natural ecosystems^4–6^.

Volcanic seeps can provide natural analogues for the effects of ocean acidification on the structure of marine ecosystems because they expose entire communities to a lifetime of elevated CO_2_ levels. Use of CO_2_ seeps in ocean acidification research has steadily increased over the past decade, with suitable sites now located across temperate^7–10^, sub-tropical^11^, and tropical ecosystems^12,13^. Most marine organisms have a planktonic stage in their life history, so recruitment into high CO_2_ seep sites will occur from populations that are not genetically adapted to future ocean acidification conditions. Therefore, the communities in the CO_2_ seep sites that become established will be comprised of organisms that are able to tolerate higher CO_2_ and lower carbonate saturation states for their entire lives (including those that live for many years). For clonal organisms, multiple generations of asexually reproducing individuals are exposed over periods of years, such as bryozoans and corals^14,15^, and seagrasses^8,16^. A few marine organisms have very limited larval dispersal, and there is evidence that populations of polychaetes at CO_2_ seeps are genetically distinct^17^, and that populations of molluscs that hatch benthic larvae have adapted to chronic ocean acidification over multiple generations through dwarfism^18^.

Since the beginning of the industrial era, atmospheric CO_2_ has increased from ~280 µatm to present day levels of 400 µatm^1^, and yet our understanding of the effects of ocean acidification that may have already occurred is limited; with most research focussed on potential impacts over the coming century. A recent study restored the carbonate chemistry saturation state of a coral reef flat to near pre-industrial levels and demonstrated that present-day net community calcification of coral reefs is already impaired by ocean acidification^19^. Due to the influence of the northward flowing Kuroshio Current^20,21^ our study region in Japan has naturally low levels of surface seawater *p*CO_2_ (280–320 µatm), which are near pre-industrial levels based on the global average (280 µatm; ref.^1^). This particular chemical setting could therefore provide information on how the increase of CO_2_ since the pre-industrial period has already affected ecosystems in other parts of the world.

The presence of ecosystem engineers can modify habitats, promote spatial complexity and facilitate the presence of other species^22,23^. Ocean acidification-driven changes to these habitat-forming organisms may therefore interact with the direct effects on those species residing in the habitat, and lead to lower species diversity in coral reefs, mussel beds and macroalgal habitats^5^. Previous studies in CO_2_ seeps have demonstrated consistent patterns of ocean acidification impacts on the structure of marine ecosystems, with the observed ecological shifts in the acidified conditions showing a reorganisation of the community including reduced biodiversity^8,24,25^, and habitat loss (as well as structural complexity)^5,11,12^. It is currently unclear, however, how those communities located at the boundaries of biogeographic regions are likely to respond to ocean acidification; in these regions many species overlap at their range margins and may demonstrate reduced fitness and performance relative to their range centre. Here, we investigate the effects of ocean acidification at a biogeographic transition zone on the Pacific coast of Japan. This region is a global biodiversity hotspot^26,27^ where temperate and subtropical communities overlap, with the co-existence of both canopy-forming fleshy macroalgae and zooxanthellate scleractinian corals. These two groups are key habitat-forming species that provide a complex three-dimensional structure that sustains a diverse ecosystem. Such a location will therefore provide information on the effects of ocean acidification on range limits at subtropical−temperate transition zones globally.

In the present study, we assessed the effects of chronic exposure to ocean acidification on intertidal and subtidal communities around a set of volcanic CO_2_ seeps off Shikine Island, Japan. We examined the community composition of benthic marine life at sites with reference levels of 300 µatm *p*CO_2_ (near pre-industrial levels) and compared them with areas exposed to increasing levels of carbon dioxide gradually up to end-of-the-century *p*CO_2_ conditions to provide the first chemical and ecological assessment of the impact of ocean acidification at a subtropical−temperate transition zone.

## Methods

### Study Site and Carbonate Chemistry

**Figure 1:**
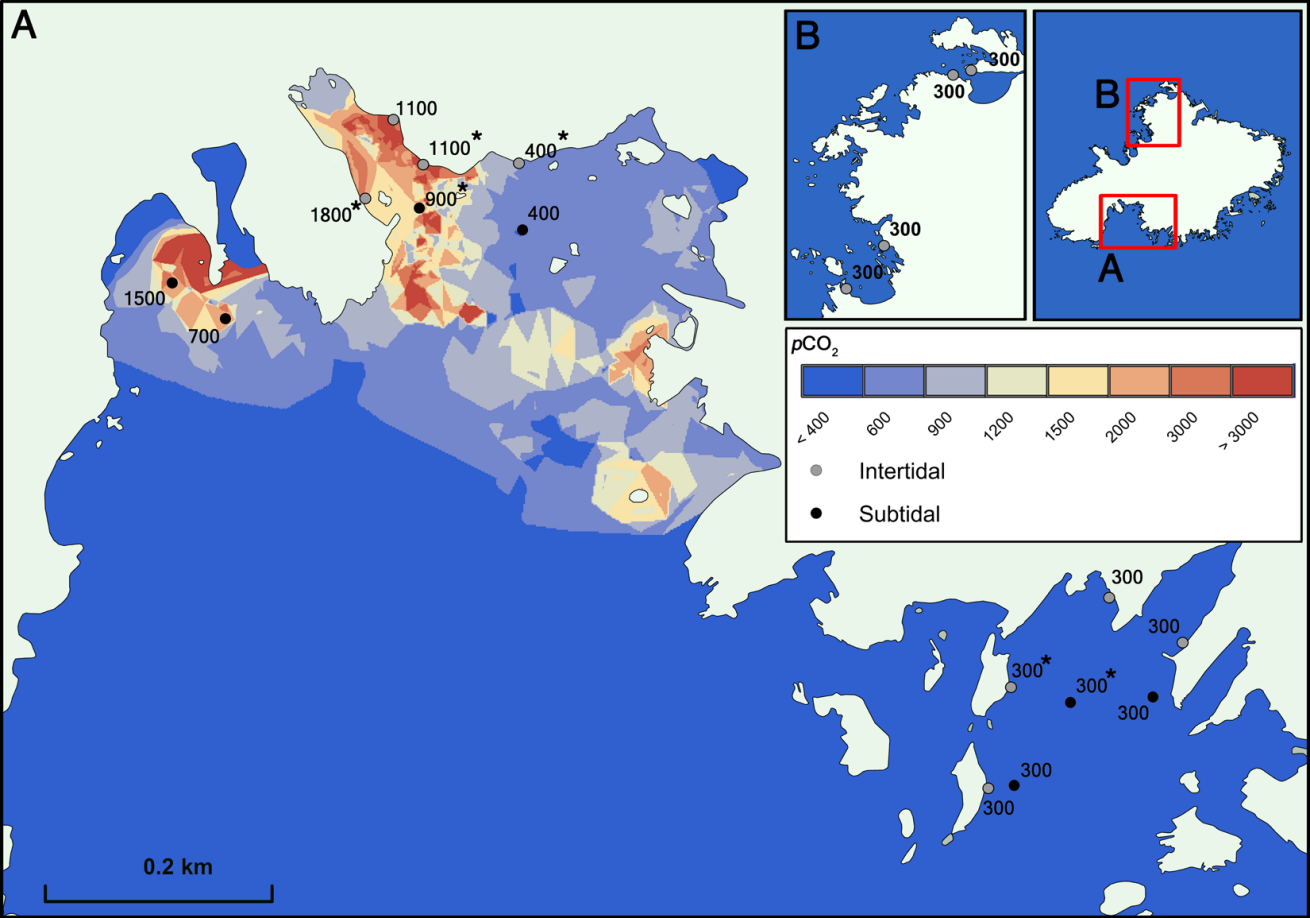
Study area (Shikine-Jima, Japan) showing intertidal and subtidal stations, and the spatial variability in *p*CO_2_. The spatial distribution of *p*CO_2_ was computed using the nearest neighbour algorithm in ArcGIS 10.2 software (http://www.esri.com/software/arcgis/). ‘*’Indicates sites where 24-hour measurements of carbonate chemistry were taken.

Shikine is a volcanic island east of the Izu peninsula in Japan (34°19′9″ N, 139° 12′18″ E) with many CO_2_ seeps in shallow waters that we surveyed using RV Tsukuba II. Different stations in the intertidal and subtidal zones (3–6 m below Chart datum) were surveyed and given a classification based on their mean *p*CO_2_ levels. Those stations with a similar *p*CO_2_ level were then grouped together for subsequent analysis. The groupings used were 300, 400, 1100 and 1800 µatm *p*CO_2_ for the intertidal, and 300, 400, 700, 900, and 1500 µatm *p*CO_2_ for the subtidal. The intertidal stations included eight ‘300 µatm’ stations, one ‘400 µatm’ station, two ‘1100 µatm’ stations and one ‘1800 µatm’ station. The subtidal included three ‘300 µatm’ stations, one ‘400 µatm’ station, one ‘700 µatm’ station, one ‘900 µatm’ station and one ‘1500 µatm’ station (see Fig. 1 for station locations). The abundance and distribution of rocky shore communities varies greatly in both space and time, even along the same stretch of coast^28^. Following the suggestion of refs^29,30^ we used multiple reference stations (eight ‘300 µatm’ *p*CO_2_ in the intertidal, and three ‘300 µatm’ *p*CO_2_ in the subtidal) to assess the variability of ‘normal’ rocky shore communities to compare with our high CO_2_ stations.

To describe the carbonate chemistry of the survey stations, pH, temperature, salinity and total alkalinity (TA) were measured through *in situ* measurements and/or discrete sampling at the respective stations using both a YSI sensor (YSI Pro Plus, USA) and a TOA-DKK multisensor (WQ-22C, TOA-DKK, Japan) in June 2015. Intertidal stations were surveyed by fixing the sensors to the shore (50 cm below the low water mark), with discrete samples collecting surface water. Subtidal stations were surveyed by fixing the sensors to the seafloor at 5–6 m depth. Discrete samples in the subtidal surveys were taken by SCUBA divers close to the bottom (5–6 m depth). Long term monitoring of pH, temperature, conductivity and dissolved oxygen of the bottom water at the ‘300 µatm’ and ‘900 µatm’ *p*CO_2_ stations was carried out in June 2016 using durafet pH sensors (Seafet, Sea-Bird Scientific, Canada) calibrated on the total scale, Hobo conductivity loggers (U24-002-C) and Hobo dissolved oxygen data loggers (U26-001) (Bourne, Onset, USA) with the sensors fixed 30 cm above the seafloor at a depth of 5–6 m. A Horiba multi-parameter meter (U-5000G, Horiba Ltd, Kyoto Japan) coupled with a GPS (eTrex30x, Garmin) was used to document the spatial variation in carbonate chemistry, where we mapped the spatial distribution of *p*CO_2_ using the nearest neighbour interpolation algorithm in ArcGIS (ESRI, New York, USA), see Fig. 1.

All pH meters, with the exception of the factory calibrated SeaFET sensors, were regularly calibrated to the NBS pH scale using three buffers 4.01, 7.00 and 10.01 (Thermo Scientific, USA). Total alkalinity of the intertidal stations was measured at the ‘300 µatm’ (n = 6), ‘400 µatm’ (n = 4), ‘1100 µatm’ (n = 4) and ‘1800 µatm’ (n = 3) stations. For the subtidal zone, bottom water samples were collected for TA measurements at the ‘300 µatm’ (n = 10), ‘400 µatm’ (n = 3), ‘700 µatm’ (n = 11), ‘900 µatm’ (n = 14), and ‘1500 µatm’ (n = 14) stations. Water samples were immediately filtered at 0.45 μm using disposable cellulose acetate filters (Dismic, Advantech, Japan) and stored at room temperature in the dark until measurement. TA was measured by titration (785 DMP Titrino, Metrohm) with HCl at 0.1 mol l^−1^, and then calculated from the Gran function between pH 4.2 and 3.0. The titrations were cross-validated using a working standard (SD: ± 9 μmol kg^−1^) and against certified reference material purchased from the A.G. Dickson laboratory. Carbonate chemistry parameters were calculated using the CO_2_SYS software^31^. Measured pH_NBS_, TA, temperature and salinity were used as the input variables, alongside the disassociation constants from Mehrbach *et al.*^32^, as adjusted by ^Dickson *et al.*33^, KSO_4_ using ^Dickson34^, and total borate concentrations from Uppström^35^.

### Biological Survey

Percent cover of intertidal macroflora and encrusting fauna were visually assessed using 25 × 25 cm quadrats. Other sessile macroinvertebrates were counted in 50 × 50 cm quadrats. In both cases, 10–15 replicate quadrats were haphazardly deployed at least a metre apart on steeply sloping rock faces at each station. Macroalgae were grouped as: ‘crustose coralline algae’, ‘non-calcareous encrusting algae’ (such as Peyssonneliaceae spp.) and ‘fleshy algae’ (typically < 5 cm in height such as corticated *Ishige okamurae*, filamentous *Chaetomorpha spiralis*, and foliose *Ulva* spp.). Some fauna were surveyed as % cover, *viz*. ‘sponges’, ‘hard corals’, ‘barnacles (>1 cm)’, ‘barnacles (<1 cm)’, ‘anemones’ and ‘colonial ascidians’. Groups of invertebrates that were counted as numbers of individuals were ‘serpulids’, ‘spirobids’, ‘mussels’, ‘oysters’, ‘chitons’, ‘carnivorous gastropods’, ‘herbivorous gastropods’ and ‘decapods’. The community structure of shallow subtidal rock was assessed by SCUBA diving using haphazardly placed 50 × 50 cm digital photoquadrats (*n* = 8–11) at the seven stations of ‘300 µatm’, ‘400 µatm’, ‘700 µatm’, ‘900 µatm’, and ‘1500 µatm’ *p*CO_2_ levels. Photographs were analysed using ImageJ^36^ by overlaying 64 points on a grid, and recording the features and organisms at each point. Coverage in the photoquadrats was grouped as follows: ‘canopy-forming algae’ (5–50 cm in height), ‘low-profile algae’ (<5 cm in height), ‘turf algae’ (filamentous algae and microalgae), ‘non-calcareous encrusting algae’, ‘branched coralline algae’, ‘crustose coralline algae’, ‘hard corals’, ‘soft corals’ and ‘rock’.

In order to assess differences in species richness between the CO_2_ levels (since taxonomic groups were used to evaluate community changes across stations), species diversity was assessed during 30-minute searches in the intertidal zone at a ‘300 µatm’ station and a ‘1100 µatm’ station. Species richness in the subtidal zone at ‘300 µatm’, ‘400 µatm’ and ‘900 µatm’ stations was assessed by identifying the different species observed in the photoquadrats. The taxonomic groups that were assigned to the species observed in the intertidal and subtidal zones are shown in Tables S1 and S2.

For the statistical analyses, the stations were grouped based on their mean *p*CO_2_ classification (see ‘Methods: Study Site and Carbonate Chemistry’). These groups were: ‘300 µatm’, ‘400 µatm’, ‘1100 µatm’ and ‘1800 µatm’ in the intertidal zone, and ‘300 µatm’, ‘400 µatm’, ‘700 µatm’, ‘900 µatm’ and ‘1500 µatm’ in the subtidal zone. Taxonomic groups were individually compared across CO_2_ levels using the Kruskal-Wallis test, with Fisher’s least significant difference (Bonferroni-adjusted) as a *post-hoc* test (statistical significance tested at *p* < 0.05; with results for individual taxonomic groups presented in full in Table S3). All statistical analyses were performed using R software (Version 3.2.4) with the Kruskal function (agricolae package).

The variation in habitat complexity along *p*CO_2_ gradients was assessed based on the abundance of sessile taxa, along with a rank (between 0 and 5) for that taxon representing the biogenic habitat complexity provided. These ranks were: Minimum habitat complexity = 0, e.g. all encrusting algae; Very low complexity = 1, e.g. small spirorbids and small barnacles; Low complexity = 2, e.g., turf and low-profile fleshy algae, sponges and non calcifying anthozoans; Moderate complexity = 3, e.g. branched coralline algae and sparse oysters; High complexity = 4, e.g. canopy-forming algae, clumps of mussels; Exceptionally high habitat complexity = 5, e.g. hard corals (see Supporting Information, Table S3). The habitat complexity was calculated as follows: the abundance of each taxonomic group was normalised (using the decostand function, vegan package) and then had the rank (0–5 score) applied, providing a habitat complexity score for each quadrat. In order to provide a relative measure across the *p*CO_2_ sites, the habitat complexity score was normalised to between 0 and 1, where the quadrat showing the maximum complexity had a score of 1. These scores were then used to calculate the mean habitat complexity and its variability (standard error) for the different *p*CO_2_ stations.

## Results

### Seawater carbonate chemistry

**Figure 2:**
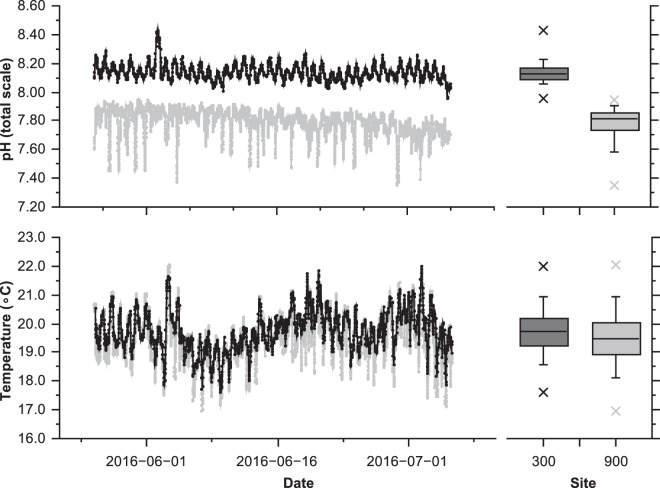
Variation of temperature and pH (total scale) over the month of June 2016 at a subtidal control site (‘300 µatm’) and a subtidal elevated CO_2_ site (‘900 µatm’). Measurements were carried out with the SeaFETs sensors deployed just above the seafloor.

There were two areas with permanently high *p*CO_2_ located in the northern part of the bay where CO_2_ was bubbling up through the seabed (Fig. 2). Our stations closest to these seeps had mean *p*CO_2_ 1773 ± 1487 µatm (‘1800 µatm’, intertidal) and 1552 ± 540 µatm (‘1500 µatm’, subtidal) (Figs 1, S1 and S2; Table 1). Variations in *p*CO_2_ over time were greatest at the highest *p*CO_2_ stations, the 10^th^ and 90^th^ percentiles are shown in Table 1 and boxplots showing the variation ranges of each parameters are shown in Fig. 2 and Supplementary Figs S1 and S2. These high levels of *p*CO_2_ corresponded to mean Ω_A_ values of 1.33 ± 0.67 at station ‘1800 µatm’ and 0.94 ± 0.33 at station ‘1500 µatm’, with minimum levels of Ω_A_ observed being 0.30 and 0.60, respectively. Farther from the seep sites, intertidal stations had mean *p*CO_2_ levels of 1182 ± 672 µatm (station ‘1100 µatm’, intertidal) and subtidal stations with mean *p*CO_2_ levels of 888 ± 471 µatm (station ‘900 µatm’, subtidal) and 714 ± 20 µatm (stations ‘700 µatm’, subtidal) (Figs 1, S1 and S2; Table 1). At stations with mean *p*CO_2_ levels of 419 ± 82 µatm (station ‘400 µatm’, intertidal) in the intertidal and 460 ± 40 µatm in the subtidal (stations ‘400 µatm’) variability in ocean acidification conditions was lower, which corresponded to a mean Ω_A_ of 2.49 ± 0.35 intertidally and 2.23 ± 0.31 subtidally. Stations far from the seeps, had stable levels of *p*CO_2_ with 299 ± 51 µatm station ‘300 µatm’, intertidal) and 342 ± 26 µatm station ‘300 µatm’, subtidal), where the mean Ω_A_ recorded were 3.27 ± 0.37 and 2.75 ± 0.14, respectively (Fig. 1; Table 1). Monitoring of the subtidal pH and temperature for over a month-period showed an average mean pH_T_ of 8.14 ± 0.06 at a subtidal ‘300 µatm’ station and an average mean pH_T_ of 7.79 ± 0.10 at the subtidal ‘900 µatm’ station (Figs 2 and S3). The ‘300 µatm’, ‘400 µatm’ and ‘900 µatm’ (‘1100 µatm’) stations represent near pre-industrial, present-day, and end of century conditions, respectively. Abiotic parameters such as dissolved oxygen, total alkalinity and depth did not differ across sites. The detailed carbonate chemistry of the surface and subtidal waters is presented in Table 1. Continuous measurements of seawater chemistry for long periods of time at stations marked with “*” in Fig. 1 are presented in Figs 2, S1, S2 and S3.

**Table 1:** Carbonate chemistry in subtidal and intertidal waters off Shikine Island, Japan.

Station	pH_NBS_	Temp. (°C)	Salinity (psu)	TA (μmol kg^−1^	*p*CO_2_ (μatm)	DIC (μmol kg^−1^)	HCO_3_^−^(μmol kg^−1^)	CO_3_^2−^ (μmol kg^−1^)	Ωcalcite	Ωaragonite
Intertidal										
300	8.27 ± 0.06	19.4 ± 0.5	34.03 ± 0.02	2237 ± 1	298.6 ± 51.0	1938.9 ± 36.3	1719.6 ± 58.1	209.4 ± 23.5	5.04 ± 0.57	3.27 ± 0.37
	(8.15–8.35)	(18.8–20.1)	(34.00–34.05)	(2236–2238)	(237.1–382.6)	(1888.4–1991.6)	(1638.4–1803.2)	(175.7–242.3)	(4.23–5.84)	(2.74–3.79)
400	8.15 ± 0.07	17.5 ± 1.1	33.97 ± 0.09	2250 ± 2	419.2 ± 82.3	2028.1 ± 35.9	1853.0 ± 55.1	160.5 ± 22.4	3.86 ± 0.54	2.49 ± 0.35
	(8.05–8.22)	(16.4–19.0)	(33.86–34.04)	(2248–2252)	(343.7–536.9)	(1985.6–2081.3)	(1786.5–1934.7)	(127.4–187.6)	(3.06–4.51)	(1.97–2.92)
1100	7.81 ± 0.22	18.5 ± 0.7	33.97 ± 0.03	2282 ± 31	1181.5 ± 672.4	2186.8 ± 82.4	2054.4 ± 103.6	92.3 ± 42.1	2.22 ± 1.01	1.44 ± 0.66
	(7.50–8.07)	(17.6–19.5)	(33.94–34.01)	(2244–2326)	(518.1–2171.2)	(2089.9–2292.9)	(1929.3–2175.8)	(43.0–143.1)	(1.03–3.44)	(0.67–2.22)
1800	7.70 ± 0.30	21.3 ± 0.4	34.30 ± 0.21	2261 ± 3	1773.4 ± 1487.0	2192.6 ± 105.4	2052.5 ± 105.7	84.5 ± 42.9	2.04 ± 1.03	1.33 ± 0.67
	(7.22–8.02)	(20.5–21.7)	(34.00–34.46)	(2259–2263)	(601.6–4308.8)	(2074.6–2360.2)	(1915.9–2199.0)	(25.1–140.0)	(0.60–3.37)	(0.39–2.20)
Subtidal										
300	8.22 ± 0.03	16.5 ± 0.0	34.51 ± 0.03	2260 ± 3	341.5 ± 26.1	2007.6 ± 14.9	1816.6 ± 22.9	178.8 ± 9.0	4.28 ± 0.22	2.75 ± 0.14
	(8.19–8.25)	(16.5–16.5)	(34.50–34.51)	(2256–2263)	(311.1–372.4)	(1989.0–2026.2)	(1790.1–1844.4)	(167.8–189.6)	(4.01–4.53)	(2.58–2.92)
400	8.11 ± 0.02	18.1 ± 0.2	34.75 ± 0.06	2252 ± 3	459.5 ± 39.9	2054.9 ± 28.7	1893.8 ± 45.4	144.3 ± 19.1	3.45 ± 0.46	2.23 ± 0.31
	(8.08–8.13)	(17.8–18.4)	(34.69–34.80)	(2249–2256)	(431.4–503.1)	(2036.5–2066.4)	(1865.3–1911.3)	(137.3–156.1)	(3.28–3.73)	(2.12–2.41)
700	7.94 ± 0.01	16.3 ± 0.1	34.54 ± 0.05	2272 ± 4	714.3 ± 20.2	2145.3 ± 4.3	2015.7 ± 5.7	103.8 ± 2.5	2.48 ± 0.06	1.60 ± 0.04
	(7.92–7.96)	(16.2–16.3)	(34.50–34.60)	(2269–2276)	(686.2–743.0)	(2139.5–2150.1)	(2007.6–2021.8)	(100.5–107.3)	(2.40–2.57)	(1.54–1.65)
900	7.90 ± 0.17	19.0 ± 0.7	34.47 ± 0.06	2270 ± 2	888.4 ± 471.3	2140.5 ± 61.2	2002.9 ± 78.7	108.0 ± 31.9	2.59 ± 0.76	1.68 ± 0.49
	(7.69–8.09)	(18.3–19.8)	(34.40–34.50)	(2267–2271)	(490.5–1382.8)	(2067.5–2219.7)	(1902.2–2108.5)	(65.3–148.8)	(1.57–3.57)	(1.02–2.31)
1500	7.66 ± 0.15	17.5 ± 0.2	34.54 ± 0.05	2248 ± 10	1552.1 ± 539.5	2212.7 ± 49.5	2097.7 ± 52.4	60.9 ± 21.2	1.46 ± 0.51	0.94 ± 0.33
	(7.47–7.87)	(17.2–17.6)	(34.50–34.60)	(2238–2263)	(855.8–2302.2)	(2142.6–2271.8)	(2020.8–2153.1)	(38.5–92.0)	(0.92–2.21)	(0.60–1.42)

pH_NBS_, temperature, salinity, and total alkalinity (TA) are measured values. Seawater *p*CO_2_, dissolved inorganic carbon (DIC), bicarbonate (HCO_3_^−^), carbonate (CO_3_^2−^), saturation states for calcite (Ωcalcite) and aragonite (Ωaragonite) are values calculated using the carbonate chemistry system analysis program CO2SYS. Values are presented as mean ± S.D. with 10^th^ and 90^th^ percentiles.

### Intertidal communities

**Figure 3:**
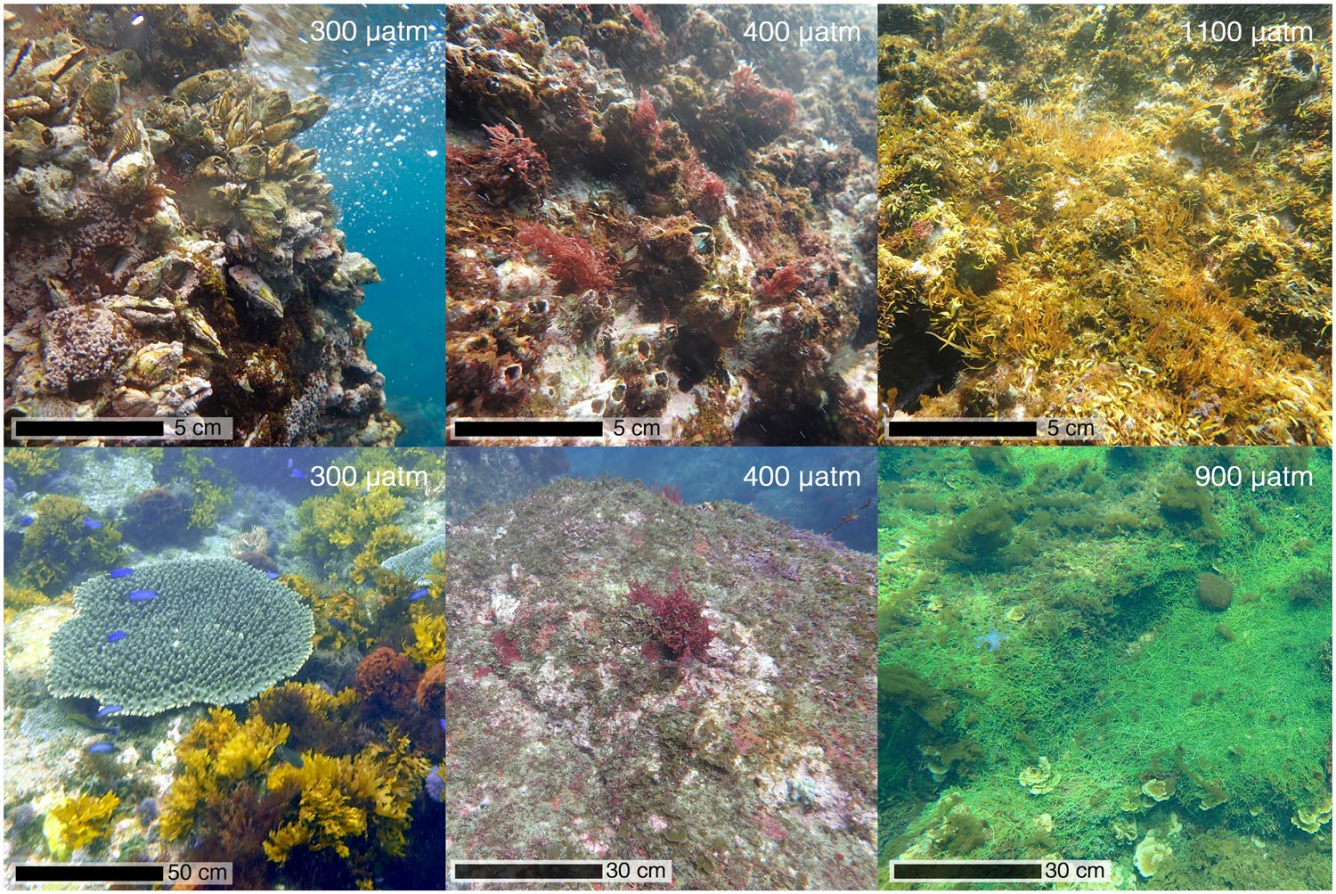
Representative ecological communities at increasing *p*CO_2_ levels. The top panels represent intertidal communities associated with mean levels of 300, 400 and 1100 µatm *p*CO_2_. The bottom panels represent subtidal communities associated with mean levels of 300, 400 and 900 µatm *p*CO_2_.

**Figure 4:**
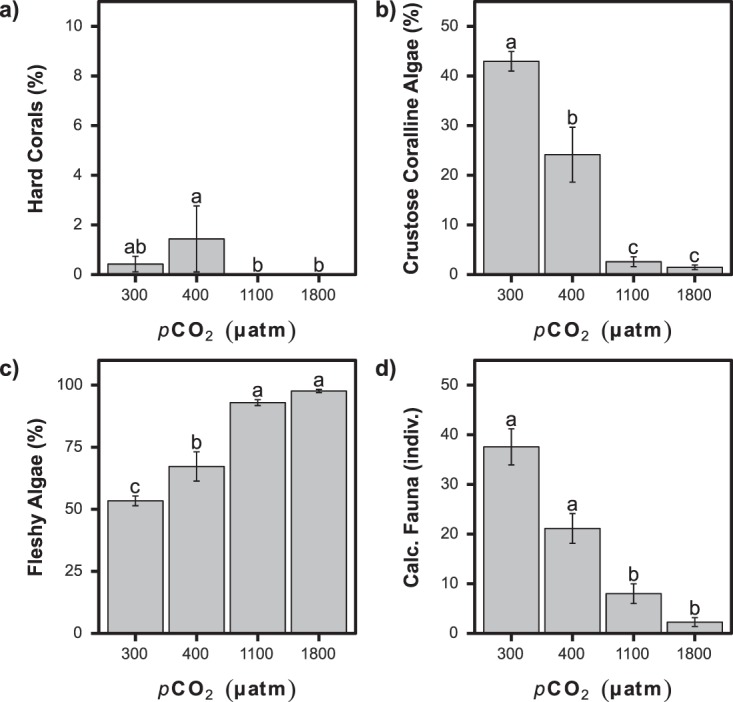
Changes in abundance (mean ± SE) of taxa (mean ± SE) with increasing *p*CO_2_ for the intertidal habitat. (**a**) Hard corals. (**b**) Coralline algae. (**c**) Fleshy algae. (**d**) Calcified fauna. A significant difference between *p*CO_2_ groups is indicated with a different letter (Kruskal-Wallis with Bonferroni-adjusted Fisher’s least significant difference).

The rocky shores of Shikine Island were characterised by thick biogenic carbonate crusts formed by coralline algae, serpulids, barnacles and molluscs with an overgrowth of fleshy algae on the low shore in the intertidal zone. There was a significant difference in the abundance of macroflora and encrusting macrofauna between the different CO_2_ levels (Figs 3 and 4). One of the most notable shifts in community composition was a very clear decrease in the coralline algae as CO_2_ levels increased; this cover fell from 43 ± 21% at ‘300 µatm’ to 24 ± 21% at 400 µatm, 3 ± 5% ‘1100 µatm’ and 1 ± 2% at ‘1800 µatm’, respectively (K-W: *H* = 99.21, *p* < 0.001; Figs 3 and 4a). There was also a significant decrease in sponge cover as CO_2_ levels increased (K-W: *H* = 19.27, *p* < 0.001; Fig. S4). Despite reductions in coralline algae and sponges, the amount of rock covered solely by biofilm remained low throughout the CO_2_ gradient, with 2 ± 6% at ‘300 µatm’, 4 ± 6% at ‘1100 µatm’ and 1 ± 2% at ‘1800 µatm’ CO_2_ (K-W: *H* = 11.46, *p* < 0.01; Fig. S4). Fleshy algae increased in abundance from 53 ± 21% at ‘300 µatm’ to 98 ± 3% at ‘1800 µatm’ (K-W: *H* = 47.57, *p* < 0.001; Fig. 3c), and non-calcareous encrusting algae increased from 4 ± 7% at ‘300 µatm’ to 18 ± 27% and 11 ± 27%, at ‘1100 µatm’ and ‘1800 µatm’, respectively (K-W: *H* = 16.77, *p* < 0.001; Fig. S4).

Most of the macrofauna investigated showed significant changes in abundance along the gradients in CO_2_. The abundance of calcifying organisms decreased as *p*CO_2_ levels rose with a significant difference between the ‘300 µatm’ and ‘400 µatm’ versus the ‘1100 µatm’ and ‘1800 µatm’, with the number of individuals falling from 38 ± 38 individuals at ‘300 µatm’ to 2 ± 3 individuals at ‘1800 µatm’ (K-W: *H* = 63.73, *p* < 0.001; Figs 3 and 4d). The abundance of the larger barnacles (>1 cm in diameter including *Megabalanus volcano* and *Megabalanus rosa*) fell from 18 ± 24 at ‘300 µatm’, 15 ± 8 at ‘400 µatm’ and 5 ± 10 individuals per 0.25 m^2^ at ‘1100 µatm’, and they were almost absent at ‘1800 µatm’ (K-W: *H* = 33.42, *p* < 0.001; Fig. S4). Mussels were also reduced in abundance from 7 ± 13 individuals at ‘300 µatm’ to 2 ± 3 individuals in the ‘1800 µatm’ (K-W: *H* = 20.80, *p* < 0.001; Fig. S4). Oysters and decapods were less common at the elevated CO_2_ stations but our surveys did not reveal statistically significant differences (K-W, *p* > 0.05) as they were not very abundant in quadrats at ‘300 µatm’ (Fig. S4). The azooxanthellate coral *Tubastrea coccinea* was observed at ‘300 µatm’ and ‘400 µatm’ but absent at ‘1100 µatm’ and ‘1800 µatm’ *p*CO_2_ (K-W: *H* = 14.32, *p* < 0.05; Fig. S4). Of the non-calcifying fauna, only sea anemones significantly increased in abundance at ‘1100 µatm’ (0.1 ± 0.4 individuals per 0.25 m^2^ at 300 µatm *vs*. 1.0 ± 1.4 at ‘1100 µatm’), they were absent from the ‘1500 µatm’ station (K-W: *H* = 53.89, *p* < 0.001; Fig. S4).

**Figure 5:**
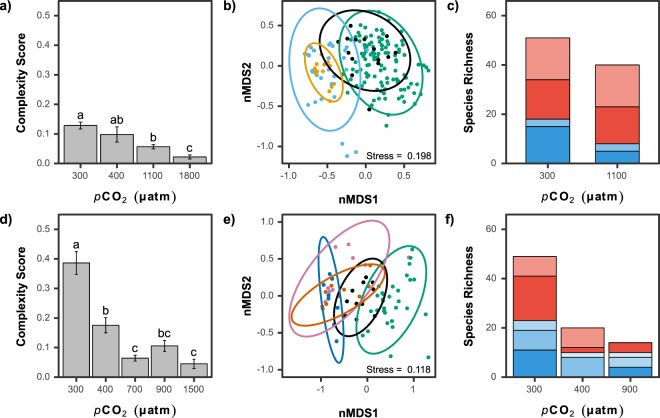
Changes in habitat complexity (mean ± SE), communities, and species richness with increasing *p*CO_2_ for intertidal (**a**–**c**) and subtidal (**d**–**f**) habitats. (**a**,**d**) A significant difference between *p*CO_2_ groups is indicated with a different letter (Kruskal-Wallis with Bonferroni-adjusted Fisher’s least significant difference). (**b**,**e**) The change in communities are illustrated by an nMDS plot based on Bray Curtis distance. The colour of each point represents the *p*CO_2_: green: ‘300 µatm’, black: ‘400 µatm’, light blue: ‘1100 µatm’ and orange: ‘1800 µatm’ for the intertidal and green: ‘300 µatm’, black: ‘400 µatm’, blue: ‘700 µatm’, red: ‘900 µatm’ and pink: ‘1500 µatm’ for the subtidal. Ellipses represent the 95% interval confidence. (**c**,**f**) Algal (blue) and faunal (red) species richness are shown with darker colours used for species only found in that site, and lighter shades for species that overlap across two sites. For the subtidal, the species overlap are graduated (from darkest to lightest) in the following order: 300–400 µatm, 300–900 µatm and 400–900 µatm (no species were common to all three sites).

Intertidal habitat complexity was provided by barnacles, mussels and oysters, and coralline algae (which formed a thick crust) at ‘300 µatm’. These calcifying groups drastically decreased in abundance with an overall shift in the communities as *p*CO_2_ levels rose (Figs 3 and 5b). These shifts lead to a decrease in the complexity of the habitat (K-W: *H* = 48.50, *p* < 0.001), reducing two-fold from ‘300 µatm’ to ‘1100 µatm’, and more than 6-fold to ‘1800 µatm’ (Fig. 5a). At the high CO_2_ levels, the main habitat was low-profile fleshy algae (Figs 3 and 5a).

Despite the increasing abundance of fleshy algae with rising *p*CO_2_, there was a 56% reduction in algal species richness from ‘300 µatm’ (18 spp.) to ‘1100 µatm’ (8 spp.) with little overlap in species composition between these two CO_2_ levels (Fig. 5c and Table S1). The ‘300 µatm’ stations had a diverse community of Rhodophyta with 16 species compared to only six species at ‘1100 µatm’. There were 33 and 32 macrofaunal taxa at ‘300 µatm’ and ‘1100 µatm’ respectively, but the community composition was very different, with only seven species common to both sets of stations (Fig. 5c and Table S1).

### Subtidal zone

**Figure 6:**
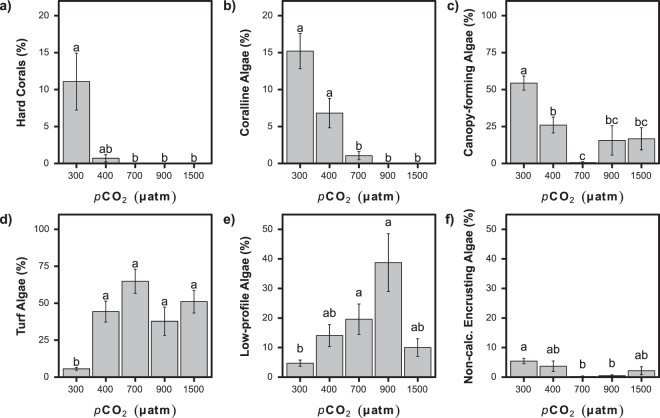
Changes in abundance (mean ± SE) of taxa with increasing *p*CO_2_ for the subtidal habitat. (**a**) Hard corals. (**b**) Coralline algae. (**c**) Canopy-forming fleshy algae. (**d**) Turf algae. (**e**) Low-profile fleshy algae. (**f**) Non-calcified encrusting algae. A significant difference between *p*CO_2_ groups is indicated with a different letter (Kruskal-Wallis with Bonferroni-adjusted Fisher’s least significant difference).

Changes in the subtidal benthic community were remarkably similar to those observed intertidally, with reduced abundances of calcifying organisms as CO_2_ levels increased from ‘300 µatm’ to ‘400 µatm’ and again to ‘700 µatm’ and beyond (Figs 3 and 6). The cover of coralline algae (K-W: *H* = 42.46, *p* < 0.001; Fig. 6b) and hard corals (K-W: *H* = 19.67, *p* < 0.001; Fig. 6a) was significantly reduced as CO_2_ levels rose. Hard corals were common at ‘300 µatm’, where they had 11 ± 22% cover, however, they were only sporadically found at ‘400 µatm’ with just two colonies observed accounting for 0.7 ± 1.6% cover, and absent from more highly elevated CO_2_ stations (Figs 3 and 6a). Soft corals and anemones were not recorded in the elevated CO_2_ stations corresponding to the end-of-the-century projections (‘700 µatm’, ‘900 µatm’ and ‘1500 µatm’) and were rare at ‘300 µatm’ and ‘400 µatm’. Due to their low abundance at ‘300 µatm’ and ‘400 µatm’, the soft corals and the anemones did not significantly differ with CO_2_ level (K-W: *H* = 6.32, *p* = 0.18 and *H* = 5.09, *p* = 0.27 respectively) (Fig. S4).

The cover of non-calcifying macroalgae was high at all subtidal stations yet there were major shifts in community composition (Figs 3 and 6c–f). Large canopy forming macroalgae had significantly reduced abundance at ‘400 µatm’ and higher *p*CO_2_ end-of-the century projections (K-W: *H* = 18.53, *p* < 0.001, Fig. 6c) whereas low-profile algae and turf algae increased in cover as CO_2_ levels rose, with their cover significantly higher at ‘400 µatm’ and higher CO_2_ level stations compared to ‘300 µatm’ stations (K-W: *H* = 23.41, *p* < 0.001 and *H* = 44.81, *p* < 0.001 Fig. 6d,e). Due to the overall reduction in the percentage of calcified and non-calcifying macroalgae, the proportion of biofilm encrusted substrata significantly increased from 2 ± 3% to 20 ± 14% at ‘300 µatm’ and ‘1500 µatm’ respectively (K-W: *H* = 32.88, *p* < 0.001; Fig. S5).

Both hard corals and canopy forming macroalgae formed a biogenically complex habitat in the subtidal zone at ‘300 µatm’ CO_2_ (Fig. 3). The sharp decrease of these two groups lead to significantly reduced habitat complexity (K-W: *H* = 50.48, *p* < 0.001) at CO_2_ levels corresponding to the mid- (‘400 µatm’) and end-of-the-century projections (‘700 µatm’, ‘900 µatm’ and ‘1500 µatm’) (Figs 3 and 5c). The communities radically changed as *p*CO_2_ rose with distinct communities observed at each *p*CO_2_ site (Fig. 5d) with less complex low-profile and turf algae dominating at the highest *p*CO_2_ (Fig. 3).

The species richness of the benthic flora and fauna was reduced by 71% as CO_2_ levels rose, with a total of 49, 20 and 14 species at ‘300 µatm’, ‘400 µatm’ and ‘900 µatm’, respectively (Fig. 5f and Table S2). This change in faunal species richness included seven hard coral species, a sea anemone, a soft coral species and a sponge, which were only observed at ‘300 µatm’. In addition, small gastropods were abundant at ‘300 µatm’, but not at ‘400 µatm’ or ‘900 µatm’. Other mobile benthic fauna that were only found in the ‘300 µatm’ stations were sea cucumbers and coral boring serpulids and barnacles. Algal diversity was greatly reduced shifting from a diverse community with 22 species at ‘300 µatm’ to only 10 species at both the ‘400 µatm’ and ‘900 µatm’ stations, with only four species overlapping in species composition between ‘400 µatm’ and ‘900 µatm’ (Fig. 5f and Table S2). This is an underestimate of algal diversity as crustose coralline algae were counted as one taxon and most turf or very low-profile algae were not included, exceptions were larger algae such as *Lobophora variegata* and *Caulerpa chemnitzia* var*. peltata*. At ‘900 µatm’, the only abundant canopy forming macroalgae was the red algae *Grateloupia elata* (Table S2).

## Discussion

Our comparisons of intertidal and subtidal rocky reef communities along natural gradients in CO_2_ have revealed that ocean acidification is a threat to many marine organisms, as it can drive fundamental shifts in coastal marine ecosystems towards simplified, low diversity communities. Abrupt changes in subtidal and intertidal communities were revealed from present-day to near-future levels of CO_2_ (300 µatm to 400 µatm), and then again to future levels (400 µatm to 700 µatm) and beyond. In natural coastal ecosystems, mean *p*CO_2_ levels predicted for as soon as the mid-century will have periods of such low aragonite saturation and high availability of inorganic carbon that this will cause biodiversity loss driven by a decline in habitat-forming species (e.g. coralline algae, canopy-forming macroalgae, scleractinian corals, and barnacles) and an increase in low-profile and turf algae. Our observations suggest that ocean acidification will shift ecosystems at subtropical−temperate transition zones from complex calcified biogenic habitats towards less complex non-calcified habitats.

Increases in dissolved CO_2_ provide a resource for algae that cannot use bicarbonate ions for their photosynthesis^37^ and is expected to increase the prevalence of macroalgae^8,9,12,38^. The significantly increased occurrence of low-profile fleshy algae with increasing *p*CO_2_ aligns with results from other shallow marine carbon dioxide seeps. However, not all macroalgae species respond in the same manner to the effects of elevated CO_2_, with some species gaining a relative advantage over their counterparts^3^. The resulting pattern is that ocean acidification alters successional development due to competition for space by a few highly tolerant species^39,40^. The prevalence of low-profile fleshy algae in our elevated *p*CO_2_ sites may contribute towards the observed decline in canopy-forming macroalgae and corals^41^. In this context, natural analogues offer opportunities to assess competitive interactions and the effects of ocean acidification on ecological functions^40^.

The presence of highly calcified communities at all of our reference sites reflects the high carbonate saturation levels that typify this region due to naturally low background *p*CO_2_ levels^20^. At the highest CO_2_ stations the exposed shells and skeletons of calcifying organisms had visible signs of dissolution, as seen in other field studies worldwide^42–45^. The decrease in the abundance of calcifying macrofauna from our reference sites to ‘400 µatm’ sites, where even the lowest Ω_aragonite_ remains higher than values typically observed nowadays in many parts of the ocean^46,47^, suggests that ocean acidification is already impairing the growth and survival of calcifiers^19^. This is a concern and provides an insight into the effects of ocean acidification in other parts of the world that have already experienced increases in *p*CO_2_ from 300 µatm to 400 µatm during the last century since the Industrial Revolution.

Communities of zooxanthellate scleractinian corals currently thrive at high latitude (here 34° N) in East Asia due to warm, northward flowing currents which bring low *p*CO_2_, high carbonate saturated waters into the region^48^. These communities are an important reservoir of diversity for hermatypic corals, and a number of species found at our study site are endemic to the region^49^. We observed an abrupt decline in their abundance and diversity as CO_2_ levels rose and CaCO_3_ saturation state fell. Despite differences in biogeography, the major ecosystem changes we recorded along the CO_2_ gradients are broadly consistent with findings from other naturally acidified tropical coral reef settings^12,13,50^. Moreover, these patterns are comparable to those seen on tropical reefs in Florida, where present-day seasonal reductions in saturation state are contributing to reef dissolution, the die-back of scleractinians and an increase in low-profile fleshy algal growth^44,51^.

The Japanese subtropical-temperate transition zone is highly diverse due to a mix of subtropical and temperate species, which allows for the coexistence of diverse macroalgae with scleractinian zooxanthellate corals. This zone is at the leading-edge for subtropical species and the trailing-edge for temperate species, and this biogeographic boundary is likely to undergo fundamental shifts with future climate change^52,53^. With increased temperature threatening corals in the tropics^54^, it could be expected that higher latitudes will act as refugia, but this would require the loss of other ecologically important species that typically dominate these latitudes^55^. Our results support the notion that ocean acidification may constrain the shift of coral to higher latitudes^56–58^.

Biogenic complexity promotes the provisioning of habitats, allowing high levels of biodiversity to be sustained within an ecosystem^5^. Reductions in habitat complexity cause a reduction in biodiversity^5,59^. We found that as CO_2_ levels rose, there was a shift from structurally complex canopy-forming fleshy algae and corals to less complex low-profile fleshy algae and an absence of corals. This reduction in habitat complexity may have contributed towards the reduced species richness in our elevated *p*CO_2_ sites, as well as the minimal overlap in observed species among the different sites as many marine organisms rely on a particular habitat (e.g. ref.^60^). As the effect of ocean acidification could cause a simplification of the ecosystems, we can expect ocean acidification to also alter the delivery and the quality of the ecosystem services associated with these marine communities^61^ and this should be a focus of future work.

Carbon dioxide seeps are open systems that allow recruitment from outside and this hinders genetic adaptation^62^. Whilst organisms that survive at such seeps may upregulate genes to acclimate to high *p*CO_2_ levels^63^, only species with very limited genetic dispersal can be expected to evolve to cope with the local conditions^64^. The CO_2_ seep systems described in this report can nevertheless provide insights into how marine ecosystems have been changing under increased anthropogenic CO_2_ and into the near future. Thus, reference sites showed pre-industrial levels of CO_2_ and sites on the fringe of the CO_2_ gradient showed present-day and mid-century CO_2_ levels with minimum variations of these levels on short periods of time. Extreme variations of *p*CO_2_ concentrations at natural analogues are commonly observed^11,13^ and may bias the observed response of organisms^65^. Such extreme variations in *p*CO_2_ levels were also observed at the highest *p*CO_2_ sites of the Shikine CO_2_ systems, yet we also located stations with small increases and variations in *p*CO_2_ that are well suited to projected levels of ocean acidification.

In conclusion, we found that an increase in CO_2_ resulted in profound community-level changes in a biodiverse subtropical-temperate transition zone. Both intertidal and subtidal communities became highly simplified, with reduced biogenic habitat complexity and biodiversity. We highlight that ocean acidification may constrain tropical coral range expansion. Our findings suggest that a threshold for macroalgal and coral habitats at the subtropical-temperate transition zone is likely to be exceeded by 2050, with even more extreme changes expected by the end-of-the-century. Overall, ocean acidification is expected to simplify coastal marine communities throughout East Asia.

## Electronic supplementary material

Supplementary materials
