# Supplementary material for: Ocean acidification drives community shifts towards simplified non-calcified habitats in a subtropical−temperate transition zone
Source: Sci Rep. 2018 Jul 27;8:11354. doi: 10.1038/s41598-018-29251-7 (PMC6063920; doi:10.1038/s41598-018-29251-7)
Supplement: Supplementary file 1 — Supplementary materials [file 41598_2018_29251_MOESM1_ESM.docx]

# Ocean acidification drives community shifts towards simplified non-calcified habitats in a subtropical−temperate transition zone

# Supplementary Information

Sylvain Agostini^1,*^, Ben P. Harvey^1^, Shigeki Wada^1^, Koetsu Kon^1^, Marco Milazzo^2^, Kazuo Inaba^1^ and Jason M. Hall-Spencer^1,3^

^1^ Shimoda Marine Research Center, University of Tsukuba, 5-10-1 Shimoda, Shizuoka 415-0025, Japan

^2^ Dipartimento di Scienze della Terra e del Mare, University of Palermo, CoNISMa, via Archirafi 28, 90123 Palermo, Italy

^3^ Marine Biology and Ecology Research Centre, University of Plymouth, Plymouth PL4 8AA, UK.

* Email address: agostini.sylvain@shimoda.tsukuba.ac.jp (S. Agostini), Tel: (+81) 0558-22-6697

Supplementary Figures

#### Fig S1: Water chemistry of intertidal waters taken as continuous measurements (every minute, except for the station 1800 µatm, every 10 min) for up to a 24-hours on Shikine Island, Japan taken at different stations representative of the different *p*CO_2_ zones: ‘300 µatm’ (June 22^nd^ 2015), ‘400 µatm’ (June 17^th^ 2015), ‘1100 µatm’ (June 21^st^ 2015), ‘1800 µatm’ (November 11^th^ 2016). Dissolved oxygen probe was not available during the collection of the ‘1800 µatm’ data.


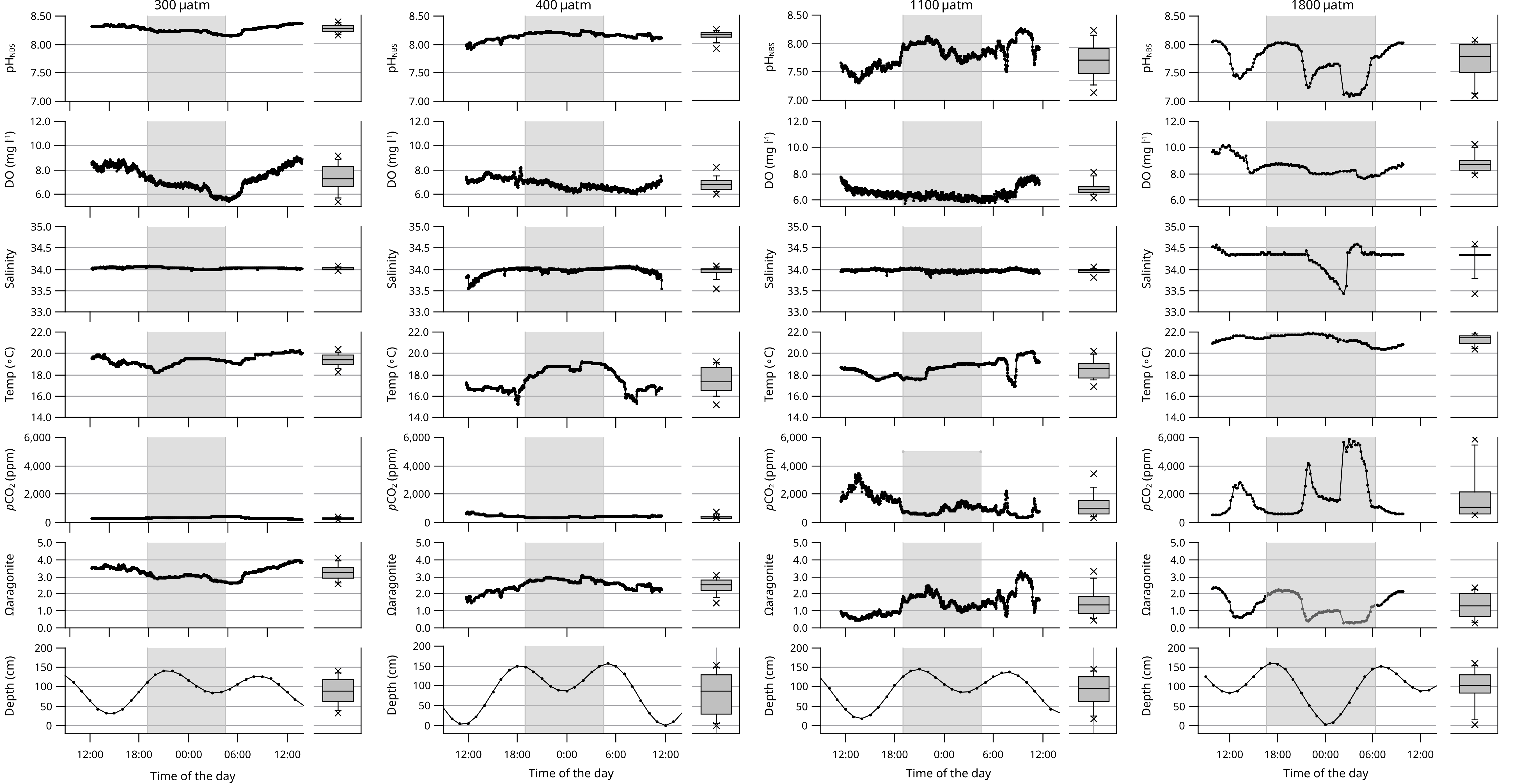


Fig. S2: Water chemistry of subtidal waters at ‘1500 µatm’ station as continuous measurements (every 5 minutes) for up to 24-hours off Shikine Island, Japan on the June 22^nd^ 2015.
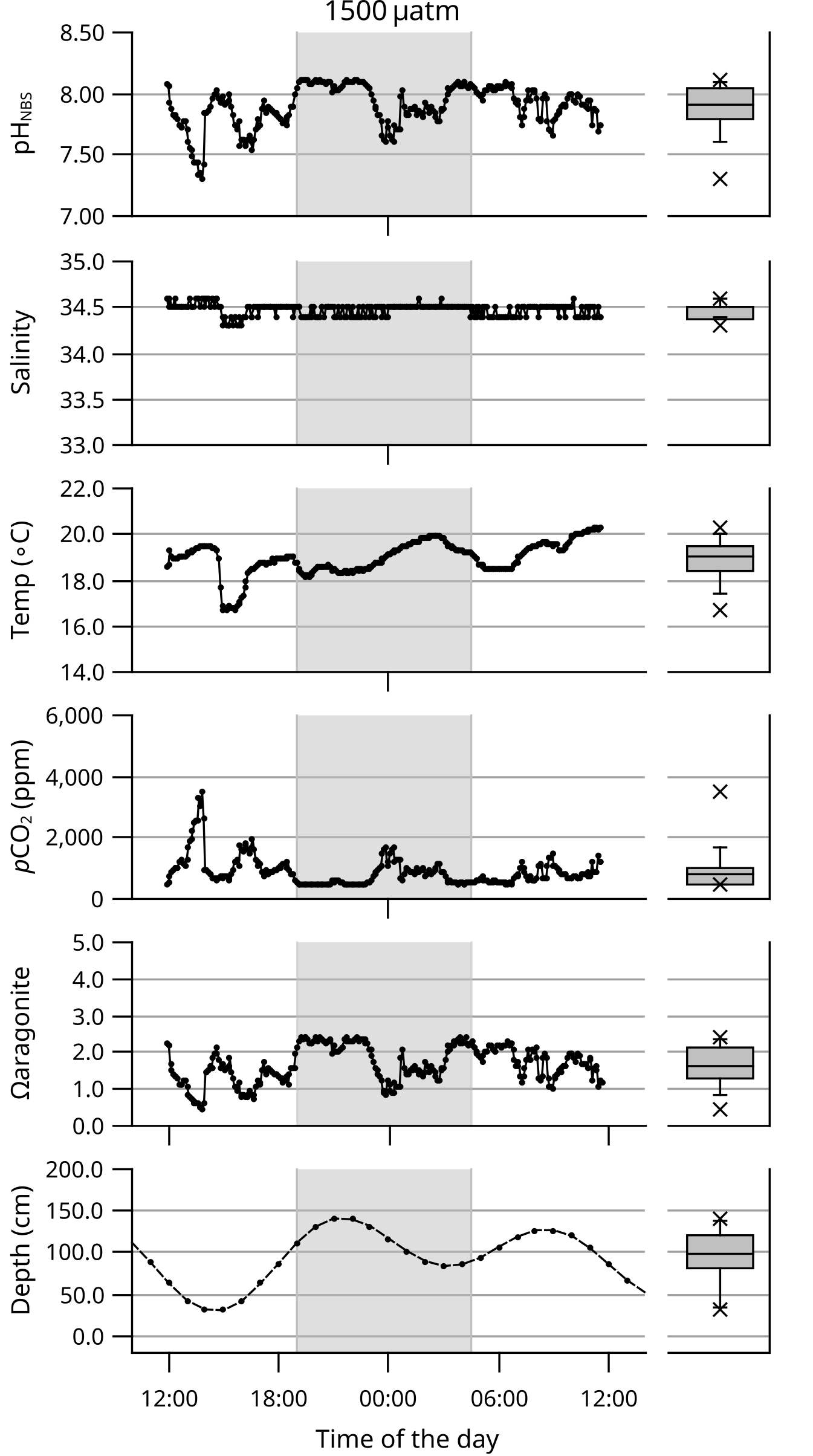


#### Fig. S3: Long term variation in water chemistry at a ‘300 μatm’ (black line) and ‘900 μatm’ (grey line) subtidal stations over the month of June 2016 (May 26^th^ 2016 to July 5^th^ 2016)

####
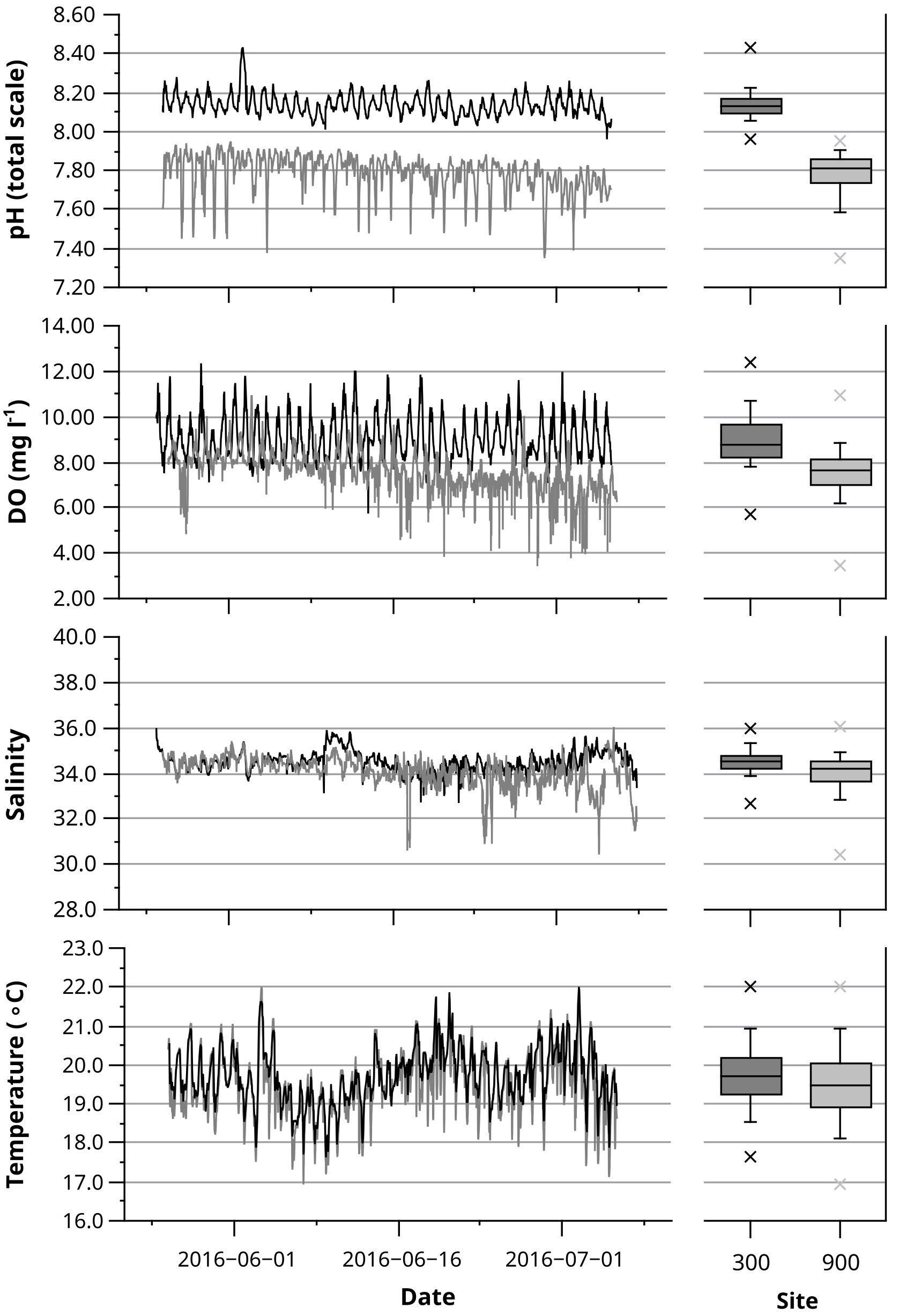


#### Fig. S4: Variation in percent cover (%) of different Taxonomic groups in the intertidal zone at stations along the CO_2_ gradient stations off Shikine Island, Japan between June 17^th^ and June 22^nd^ 2015. Letters above indicate statistical groups after multiple comparison using Kruskal-Wallis (Bonferroni-adjusted), the absence of letters signifies that the overall effect was non-significant.


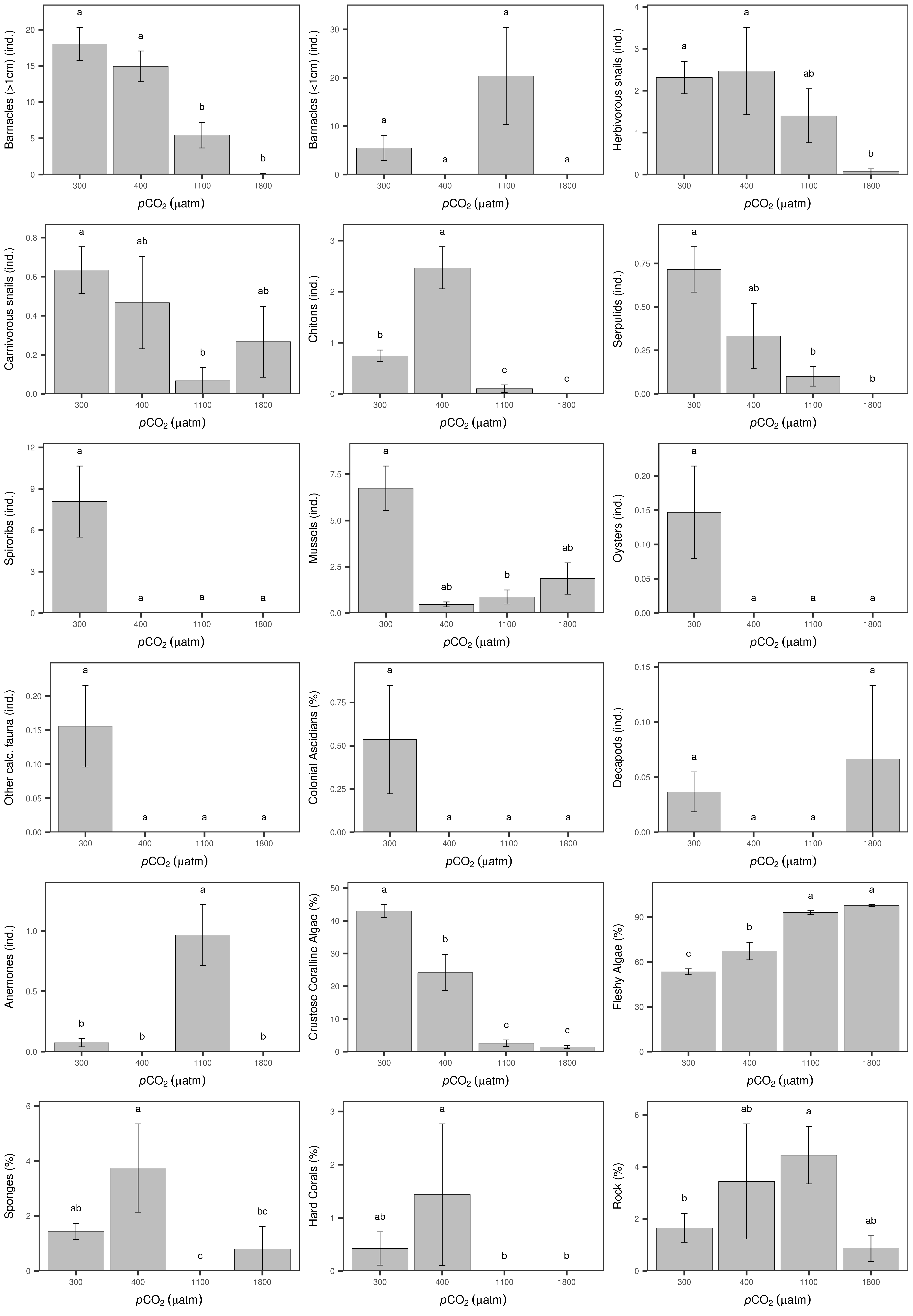


#### Fig. S5: Variation in percentage of coverage of different Taxonomic groups in the subtidal zone at stations along the CO_2_ gradient stations off Shikine Island, Japan on 1^st^ May 2015 with the exception of ‘700 µatm’ which was surveyed later on the 7^th^ July 2016. Letters above indicate statistical groups after multiple comparison using Kruskal-Wallis (Bonferroni-adjusted), the absence of letters signifies that the overall effect was non-significant (Kruskal-Wallis, p > 0.05). In addition to the data presented in the figure below, one holothurid and one sponge were counted at ‘300 µatm’ and 4 *Aplysia kurodai* were counted at the ‘1800 µatm’ site.

####
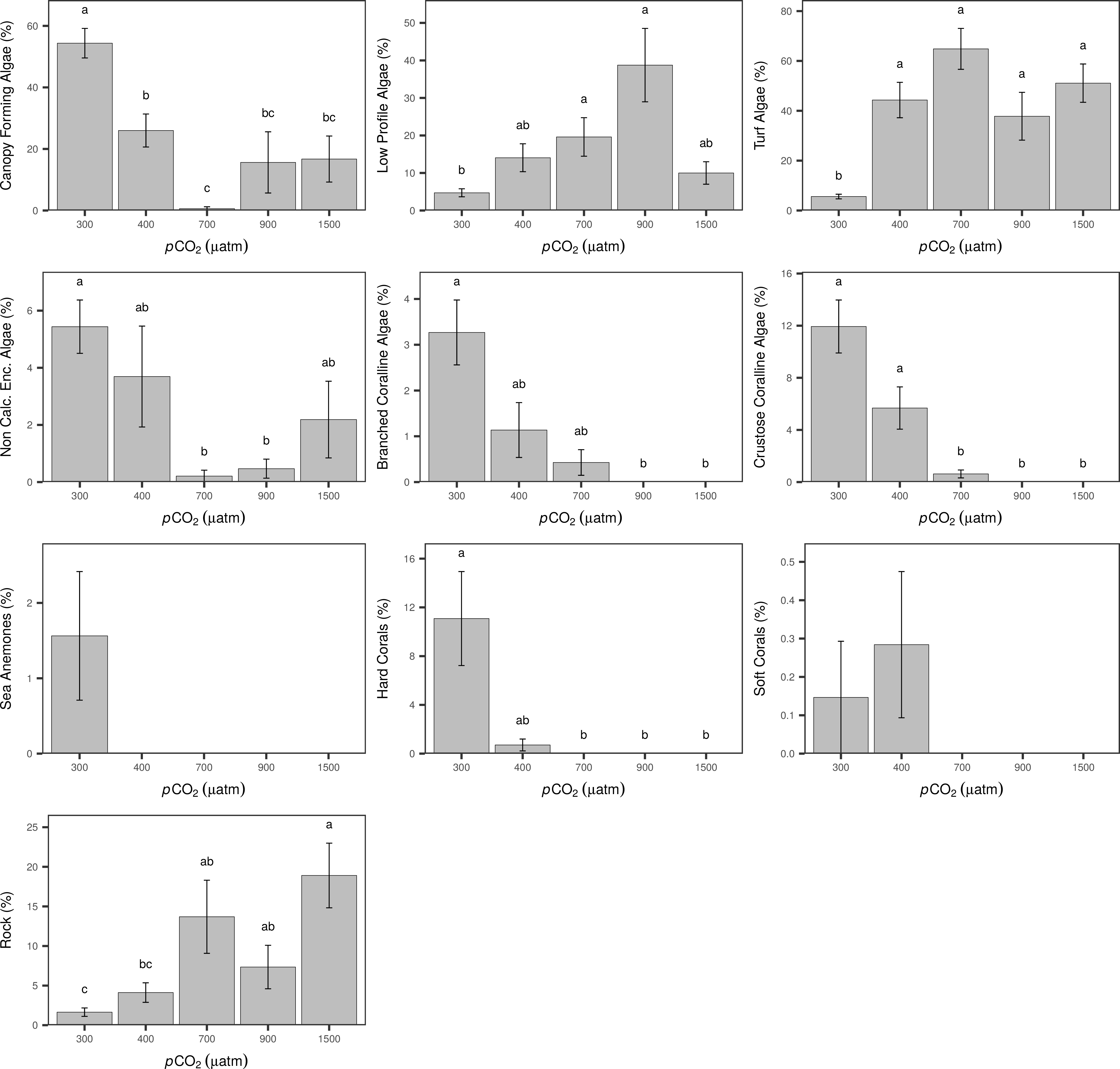


#### Table S1: Macroalgal and macrofaunal presence (dots) during a 30 min search at both 300 µatm and 1100 µatm in the intertidal at Shikine Island, Japan in June 2015. Heavily-calcified organisms are shown in bold. Total numbers of taxa at each station are given at the end of the Table.

| Taxa |  | Taxonomic Group | ‘300 µatm’ | ‘1100 µatm’ |
| --- | --- | --- | --- | --- |
| Chlorophyta | *Chaetomorpha spiralis* (Okamura, 1903) | fleshy algae |  | ● |
|  | *Ulva* (Linnaeus, 1753) sp. | fleshy algae |  | ● |
|  | *Ulva conglobata* (Kjellman, 1897) | fleshy algae | ● |  |
| Rhodophyta | *Ahnfeltiopsis concinna* ((J.Agardh) P.C. Silva & DeCew, 1992) | fleshy algae |  | ● |
|  | *Chondracanthus intermedius* ((Suringar) Hommersand, 1993) | fleshy algae | ● |  |
|  | *Chondrophycus undulatus* ((Yamada) Garbary & Harper, 1998) | fleshy algae | ● |  |
|  | *Chondrus verrucosa* (Mikami, 1965) | fleshy algae | ● |  |
|  | **Corallinaceae (Lamouroux, 1812) sp. 1** | Coralline algae | ● | ● |
|  | **Corallinaceae (Lamouroux, 1812) sp. 2** | Coralline algae | ● | ● |
|  | *Gracilaria textorii* ((Suringar) De Toni, 1895) | fleshy algae | ● |  |
|  | *Grateloupia chiangii* (S.Kawaguchi & H.W. Wang, 2001) | fleshy algae | ● |  |
|  | *Grateloupia crispata* ((Okamura) Y.-P. Lee, 2008) | fleshy algae | ● | ● |
|  | *Grateloupia crispata* ((Okamura) Y.-P. Lee, 2008) | fleshy algae |  | ● |
|  | *Grateloupia sparsa* ((Okamura) Chiang, 1970) | fleshy algae | ● |  |
|  | *Grateloupia turuturu* (Yamada, 1941) | fleshy algae | ● |  |
|  | *Hypnea variabilis* (Okamura, 1909) | fleshy algae | ● |  |
|  | *Laurencia saitoi* (L.P. Perestenko, 1980) | fleshy algae | ● |  |
|  | *Lomentaria hakodatensis* (Yendo, 1920) | fleshy algae | ● |  |
|  | *Mastocarpus stellatus* ((Stackhouse) Guiry, 1984) | fleshy algae | ● |  |
|  | Peyssonneliaceae (Denizot, 1968) sp. | Encrusting non-calcareous algae |  | ● |
|  | *Polyopes prolifer* ((Hariot) Kawaguchi & Wang, 2002) | fleshy algae |  | ● |
|  | *Pterocladiella capillacea* ((S.G. Gmelin) Santelices & Hommersand, 1997) | fleshy algae | ● |  |
|  | *Sarcodia montagneana* ((J.D. Hooker & Harvey) J.Agardh, 1852) | fleshy algae | ● |  |
| Ochrophyta | *Ishige okamurae* (Yendo, 1907) | fleshy algae | ● |  |
|  | Total number of algal taxa |  | 18 | 8 |

Table S1 continued

| Taxa |  | Taxonomic Group | ‘300 µatm’ | ‘1100 µatm’ |
| --- | --- | --- | --- | --- |
| Demospongiae | *Halichondria* (Halichondria) okadai (Kadota, 1922) | Sponges | ● | ● |
| Anthozoa | *Actinia equina* (Linnaeus, 1758) | Anemones |  | ● |
|  | Sphenopidae (Hertwig, 1882) | Other colonial organisms |  | ● |
|  | ***Tubastraea coccinea* (Lesson, 1829)** | Hard corals | ● |  |
| Polychaeta | **Spirorbinae (Chamberlin, 1919)** | Spirobids | ● | ● |
|  | **Serpulidae (Rafinesque, 1815)** | Serpulids | ● | ● |
| Polyplacophora | ***Acanthochitona achates* (Gould, 1859)** | Chitons | ● | ● |
|  | ***Liolophura japonica* (Lischke, 1873)** | Chitons | ● | ● |
|  | ***Onithochiton hirasei* (Pilsbry, 1901)** | Chitons | ● |  |
| Gastropoda | ***Patelloida saccharina* (Linnaeus, 1758)** | Herbivorous gastropods | ● | ● |
|  | ***Patelloida saccharina lanx* (Reeve, 1855)** | Herbivorous gastropods | ● | ● |
|  | ***Cellana grata* (Gould, 1859)** | Herbivorous gastropods | ● | ● |
|  | ***Cellana toreuma* (Reeve, 1854)** | Herbivorous gastropods | ● | ● |
|  | ***Lottia kogamogai* (Sasaki & Okutani, 1994)** | Herbivorous gastropods | ● |  |
|  | ***Scutellastra flexuosa* (Quoy & Gaimard, 1834)** | Herbivorous gastropods |  | ● |
|  | ***Siphonaria signa* (Reeve, 1846)** | Herbivorous gastropods |  | ● |
|  | ***Siphonaria japonica* (Donovan, 1824)** | Herbivorous gastropods | ● | ● |
|  | ***Siphonaria sirius* (Pilsbry, 1894)** | Herbivorous gastropods | ● | ● |
|  | ***Sabia conica* (Schumacher, 1817)** | Herbivorous gastropods |  | ● |
|  | ***Pictodiloma suavis* (Philippi, 1850)** | Herbivorous gastropods |  | ● |
|  | ***Drupa ricinus lischkei* (Hidalgo, 1904)** | Carnivorous gastropods | ● |  |
|  | ***Ergalatax contracta* (Reeve, 1846)** | Carnivorous gastropods | ● |  |
|  | ***Turrilatirus nagasakiensis* (E.A. Smith, 1880)** | Carnivorous gastropods | ● |  |
|  | ***Mancinella echinata* (Blainville, 1832)** | Carnivorous gastropods |  | ● |
|  | ***Mitrella burchardi* (Dunker, 1877)** | Carnivorous gastropods |  | ● |
|  | ***Tenguella granulata* (Duclos, 1832)** | Carnivorous gastropods | ● |  |
|  | ***Morula iostoma* (Reeve, 1844)** | Carnivorous gastropods | ● |  |
|  | ***Tenguella musiva* (Kiener, 1835)** | Carnivorous gastropods |  | ● |
|  | ***Morula uva* (Röding, 1798)** | Carnivorous gastropods | ● |  |
|  | ***Muricidae (Rafinesque, 1815)*** | Carnivorous gastropods | ● |  |
|  | ***Monoplex parthenopeus (Salis Marschlins, 1793)*** | Carnivorous gastropods |  | ● |
|  | ***Purpura panama (Röding, 1798)*** | Carnivorous gastropods | ● | ● |
|  | ***Reishia clavigera (Küster, 1860)*** | Carnivorous gastropods | ● | ● |
|  | ***Strigatella scutulata (Gmelin, 1791)*** | Carnivorous gastropods | ● |  |
|  | ***Reishia bronni (Dunker, 1860)*** | Carnivorous gastropods |  | ● |
|  | ***Reishia luteostoma (Holten, 1803)*** | Carnivorous gastropods |  | ● |
|  | ***Thalessa virgata (Dillwyn, 1817)*** | Carnivorous gastropods | ● |  |
|  | ***Trochus rota (Dunker, 1860)*** | Herbivorous gastropods | ● |  |
|  | ***Turbo stenogyrus (P.Fischer, 1873)*** | Herbivorous gastropods | ● |  |
|  | ***Hespererato scabriuscula (Gray, 1832)*** | Herbivorous gastropods |  | ● |
| Bivalvia | ***Brachidontes mutabilis (Gould, 1861)*** | Mussels |  | ● |
|  | ***Saccostrea kegaki (Torigoe & Inaba, 1981)*** | Oysters |  | ● |
|  | ***Saccostrea scyphophilla (Peron & Lesueur, 1807)*** | Oysters | ● |  |
| Maxillopoda | ***Capitulum mitella (Linnaeus, 1758)*** | Large barnacles (> 1cm) | ● | ● |
|  | ***Chthamalus challengeri krakatauensis (Broch, 1931)*** | Small barnacles (< 1cm) | ● | ● |
|  | ***Megabalanus rosa (Pilsbry, 1916)*** | Large barnacles (> 1cm) | ● | ● |
|  | ***Megabalanus volcano (Pilsbry, 1916)*** | Large barnacles (> 1cm) | ● |  |
|  | ***Tetraclita japonica (Pilsbry, 1916)*** | Large barnacles (> 1cm) | ● | ● |
|  | Total number of faunal taxa | | 33 | 32 |

Table S2: Macroalgal and benthic macrofaunal presence (dots) during the photo quadrat survey at the 300 µatm, 400 µatm, 900 µatm in the subtidal at Shikine Island, Japan in June 2015. Heavily-calcified organisms are shown in bold. Total numbers of taxa at each station are given at the end of the table.

| Taxa |  | Taxonomic Group | | ‘300 µatm’ | | ‘400 µatm’ | | ‘900 µatm’ | |  |
| --- | --- | --- | --- | --- | --- | --- | --- | --- | --- | --- |
| Chlorophyta | *Codium* (Stackhouse, 1797) spp. | Encrusting non-calcareous algae | | ● | | ● | |  | |  |
|  | *Codium coactum* (Okamura, 1902) | Encrusting non-calcareous algae | | ● | |  | | ● | |  |
|  | *Caulerpa chemnitzia* ((Esper) J.V.Lamououx, 1809) | Low-profile algae | |  | | ● | | ● | |  |
|  | *Ulva* (Linnaeus, 1753) spp. | Low-profile algae | |  | |  | | ● | |  |
| Rhodophyta | *Asparagopsis taxiformis* ((Delile) Trevisan de Saint-Léon, 1845) | Canopy-forming algae | | ● | | ● | |  | |  |
|  | ***Amphiroa*** (J.V. Lamouroux, 1812) sp. | Branching Coralline Algae | | ● | | ● | |  | |  |
|  | *Callophyllis crispata* (Okamura, 1896) | Canopy-forming algae | | ● | |  | |  | |  |
|  | *Chondracanthus tenellus* ((Harvey) Hommersand, 1993) | Canopy-forming algae | | ● | |  | |  | |  |
|  | *Chondria ryukyuensis* (Yamada, 1935) | Canopy-forming algae | | ● | |  | |  | |  |
|  | **Corallinaceae (Lamouroux, 1812)** | Crustose coralline algae | | ● | |  | | ● | |  |
|  | *Delisea pulchra* ((Greville) Montagne, 1844) | Canopy-forming algae | | ● | |  | | ● | |  |
|  | *Eucheuma serra* ((J.Agardh) J.Agardh, 1847) | Canopy-forming algae | | ● | |  | |  | |  |
|  | *Gelidium elegans* (Kützing, 1868) | Canopy-forming algae | | ● | |  | |  | |  |
|  | *Grateloupia angusta* ((Okamura) S.Kawaguchi & H.W.Wang, 2001) | Canopy-forming algae | | ● | |  | |  | |  |
|  | *Grateloupia* (C.Agardh, 1822) sp. | Canopy-forming algae | | ● | |  | | ● | |  |
|  | ***Lithophyllum okamurae f. japonicum*** (Foslie) | Crustose coralline algae | | ● | | ● | |  | |  |
|  | *Martensia jejuensis* (Y. Lee, 2004) | Canopy-forming algae | | ● | |  | |  | |  |
|  | *Meristotheca papulosa* ((Montagne) J.Agardh, 1872) | Canopy-forming algae | | ● | | ● | |  | |  |
|  | Peyssonneliaceae (Denizot, 1968) | Non calc. encrusting algae | | ● | | ● | |  | |  |
|  | *Pterocladiella capillacea* ((S.G. Gmelin) Santelices & Hommersand, 1997) | Canopy-forming algae | |  | |  | | ● | |  |
|  | *Ptilophora subcostata* ((Okamura) R.E.Norris, 1987) | Canopy-forming algae | | ● | |  | |  | |  |
|  | *Sarcodia ceylanica* (Harvey ex Kützing, 1869) |  | | ● | | ● | |  | |  |
|  | ***Tricleocarpa cylindrica* ((J.Ellis & Solander) Huisman & Borowitzka, 1990)** | Branched coralline algae | | ● | |  | |  | |  |
| Ochrophyta | *Dictyopteris undulata* (Holmes, 1896) | | Canopy-forming algae | |  | | ● | | ● | |
|  | *Dictyota dichotoma* ((Hudson) J.V. Lamouroux, 1809) | | Canopy-forming algae | |  | |  | | ● | |
|  | *Distromium decumbens* ((Okamura) Levring, 1940) | | Low-profile algae | | ● | | ● | |  | |
|  | *Lobophora variegata* ((J.V. Lamouroux) Womersley ex E.C. Oliveira, 1977) | | Low-profile algae | |  | |  | | ● | |
|  | *Padina arborescens* (Holmes, 1896) | | Canopy-forming algae | | ● | |  | |  | |
|  | *Rugulopteryx okamurae* ((E.Y. Dawson) I.K. Hwang, W.J. Lee & H.S. Kim, 2009) | | Canopy-forming algae | | ● | |  | |  | |
|  | Number of taxa | |  | | 23 | | 10 | | 10 | |

Table S2 continued

| Taxa |  | Taxonomic Group | ‘300 µatm’ | ‘400 µatm’ | ‘900 µatm’ |
| --- | --- | --- | --- | --- | --- |
| Demospongiae | Demospongiae (Sollas, 1885) | Other invertebrates | ● | ● |  |
| Scleractinia | ***Acropora solitaryensis* (Veron & Wallace, 1984)** | Hard corals | ● |  |  |
|  | ***Alveopora japonica* (Eguchi, (1968))** | Hard corals | ● |  |  |
|  | ***Cyphastrea* (Milne Edwards & Haime, 1848) sp.** | Hard corals | ● | ● |  |
|  | ***Dipsastraea speciosa* (Dana, 1846)** | Hard corals | ● | ● |  |
|  | ***Goniastrea complex* (Milne Edwards & Haime, 1848) sp 1** | Hard corals | ● |  |  |
|  | ***Hydnophora exesa* (Pallas, 1766)** | Hard corals | ● |  |  |
|  | ***Montipora turgescens* (Bernard, 1897)** | Hard corals | ● |  |  |
|  | ***Paragoniastrea australensis* (Milne Edwards, 1857)** | Hard corals | *●* |  |  |
|  | ***Porites heronensis* (Veron, 1985)** | Hard corals | ● |  |  |
| Actinidae | *Entacmaea quadricolor* (Leuckart in Rüppell & Leuckart, 1828) | Sea Anemone | ● |  |  |
| Nephtheidae | *Dendronephthya* (Kuekenthal, 1905) sp. | Soft corals | ● | ● |  |
| Zoanthidae | *Zoanthus* (Lamarck, 1801) sp. | Soft corals |  |  | ● |
| Phymanthidae | *Phymanthidae* (Andres, 1883) sp. | Sea Anemone |  | ● |  |
| Polychaeta | **Serpulidae (Rafinesque, 1815)** | Serpulids | ● | ● |  |
| Gastropoda | **Tegulidae (Kuroda, Habe & Oyama, 1971)** | Herbivorous gatropods | ● |  |  |
|  | **Trochidae (Rafinesque, 1815)** | Herbivorous gatropods | ● |  |  |
|  | **Muricidae (Rafinesque, 1815) sp. 1** | Carnivorous gastropods | ● |  |  |
|  | **Muricidae (Rafinesque, 1815) sp. 2** | Carnivorous gastropods | ● |  |  |
|  | **Muricidae (Rafinesque, 1815) sp. 3** | Carnivorous gastropods | ● |  |  |
|  | **Columbellidae (Swainson, 1840) sp. 1** | Carnivorous gastropods | ● | ● |  |
|  | **Columbellidae (Swainson, 1840) sp. 2** | Carnivorous gastropods | ● | ● |  |
|  | **Columbellidae (Swainson, 1840) sp. 3** | Carnivorous gastropods | ● | ● |  |
|  | **Mitridae (Swainson, 1831) sp. 1** | Carnivorous gastropods | ● |  |  |
|  | **Mitridae (Swainson, 1831) sp. 2** | Carnivorous gastropods | ● |  |  |
|  | **Neogastropoda (Wenz, 1938) sp. 1** | Carnivorous gastropods | ● |  |  |
|  | **Neogastropoda (Wenz, 1938) sp. 2** | Carnivorous gastropods |  |  | ● |
|  | *Aplysia kurodai.* (Linnaeus, 1767) | Not attributed |  |  | ● |
| Maxillopoda | **Balanomorpha (Pilsbry, 1916) sp.** | Not attributed | ● |  |  |
| Malacostraca | Decapoda (Latreille, 1802) sp. | Not attributed |  | ● |  |
| Holothuroidea | *Holothuria* (Stauropora) pervicax (Selenka, 1867) | Not attributed | ● |  |  |
| Actinopterygii | Gobiidae (Cuvier, 1816) sp. | Fishes |  |  | ● |
|  | Number of taxa |  | 26 | 10 | 4 |

Table S3: Summary table of overall *p*CO_2_ effects on organisms recorded on the rocky shores of Shikine island, Japan, including statistical analysis results (Kruskal-Wallis, *p*-value Bonferroni-corrected; *** *p* < 0.001, ** *p* < 0.01, *, *p* < 0.05). Direction of response between ‘Pre-industrial’ and ‘RCP.5’ is indicated by either an increase ‘+’, decrease ‘-’, or non-significant change ‘n.s.’. Complexity factor (scale 1 to 5) attributed to each category for calculation of the habitat complexity score shown in Figure 5.

|  | **Categories** | **Direction** | **Kruskal Wallis *H*** | **Complexity factor** |
| --- | --- | --- | --- | --- |
| **Intertidal** | Coralline Algae | - | 99.21 (***) | 2 |
|  | Fleshy Algae | + | 47.57 (***) | 2 |
|  | Non-Calcareous Encrusting Algae | + | 16.77 (***) | 0 |
|  | Sponges | - | 19.27 (***) | 2 |
|  | Colonial Ascidians | - | 9.64 (*) | 1 |
|  | Biofilm on Flat Rock | + | 11.46 (**) | 0 |
|  | Hard Corals | - | 9.81 (*) | 5 |
|  | Anemones | + | 53.39 (***) | 2 |
|  | Barnacles (> 1 cm) | - | 33.42 (***) | 4 |
|  | Barnacles (< 1 cm) | n.s. | 6.76 | 1 |
|  | Decapods | n.s. | 2.27 | 0 |
|  | Mussels | - | 20.80 (***) | 4 |
|  | Oysters | n.s. | 3.99 | 3 |
|  | Carnivorous Gastropods | - | 10.76 (*) | 0 |
|  | Herbivorous Gastropods | - | 12.50 (**) | 0 |
|  | Chitons | - | 42.11 (***) | 0 |
|  | Serpulids | - | 16.39 (***) | 2 |
|  | Spirorbids | - | 9.53 (*) | 1 |
| **Subtidal** | Hard Corals | - | 19.67 (***) | 5 |
|  | Soft Corals | n.s. | 6.32 | 2 |
|  | Anemones | n.s. | 5.09 | 2 |
|  | Turf algae | + | 44.81 (***) | 2 |
|  | Branched Coralline Algae | - | 20.96 (***) | 3 |
|  | Crustose Coralline Algae | - | 42.09 (***) | 2 |
|  | Low-Profile Algae | + | 50.13 (***) | 2 |
|  | Canopy-Forming Algae | - | 35.71 (***) | 4 |
|  | Non-Calcareous Encrusting Algae | - | 19.73 (**) | 0 |
|  | Biofilm on Flat Rock | + | 32.88 (***) | 0 |
